# A phase I safety and efficacy clinical trial of plocabulin and gemcitabine in patients with advanced solid tumors

**DOI:** 10.1007/s10637-024-01458-8

**Published:** 2024-08-03

**Authors:** Mohammad H. Ghalib, Mariano Provencio Pulla, Maria J. De Miguel Luken, Virginia Calvo de Juan, Imran Chaudhary, M Bakri Hammami, Sindhu Vikash, Radhashree Maitra, Sara Martinez, Carmen Kahatt, Sonia Extremera, Salvador Fudio, Sanjay Goel

**Affiliations:** 1 Montefiore Einstein Comprehensive Cancer Center, Bronx, NY USA; 2 Now at Rutgers Cancer Institute, New Brunswick, NJ USA; 3 Dept. Servicio de Oncología Médica, University Hospital Puerta de Hierro Majadahonda, Majadahonda, Madrid, Spain; 4 Early Phase Clinical Trial Unit, Hospital Madrid Norte San Chinarro – Centro Integral Oncologico Clara Campal, Madrid, Spain; 5 Medical Oncology Department, University Hospital Puerta de Hierro Majadahonda, Majadahonda, Madrid, Spain; 6 Clinical Development, PharmaMar, Colmenar Viejo, Madrid, S.A Spain

**Keywords:** Plocabulin, Gemcitabine, Phase I, Ovarian cancer

## Abstract

Plocabulin (Plo) induces depolymerization of tubulin fibers with disorganization and fragmentation of the microtubule network leading to mitosis. Plo combined with gemcitabine (Gem) showed synergistic anti-tumor activity in preclinical studies. This phase I trial evaluated the safety, pharmacokinetics (PK) and efficacy of Plo 10-min infusion plus Gem on Day 1 and 8 every 3-week in patients with advanced solid tumors. Fifty-seven patients were enrolled into 8 dose levels (DLs); 74%: females; 74%: ECOG performance status 1; median age: 62 years; median number of prior lines of therapy:3. Dose-limiting toxicities (DLT) in Cycle 1 were grade (G) 3 intestinal obstruction at the maximum tolerated dose (MTD), G3 peripheral sensory neuropathy (PSN), G3 abdominal pain, and G4 thrombocytopenia (1 patient each). The highest DL (DL8: Plo 10.5 mg/m^2^/Gem 1000 mg/m^2^) was the MTD. Accrual into DL7 (Plo 10.0 mg/m^2^/Gem 1000 mg/m^2^) was stopped before it was formally defined as the recommended dose (RD). Most common treatment-related adverse events (AEs) were fatigue (56%), nausea (55%), diarrhea (31%); G3/4 hematologic toxicities comprised anemia (35%), neutropenia (27%) and thrombocytopenia (17%). No treatment-related deaths occurred. PK parameters for Gem or dFdU at all DLs were in line with reference values from the literature. Six of 46 evaluable pts were responders (overall response rate:13%). Of note, 2 partial responses (PR) and 2 stable disease (SD) ≥ 4 months occurred among 13 pts with ovarian cancer. The combination of Plo and Gem is well tolerated. The MTD was Plo 10.5 mg/m^2^/Gem 1000 mg/m^2^. No PK drug-drug interaction was found. The most encouraging outcome occurred in ovarian cancer patients.

**Supplementary Information:**

The online version contains supplementary material available at 10.1007/s10637-024-01458-8.

## Introduction

Despite significant progress over the past half a century, metastatic cancers continue to have a high mortality rate, underscoring the urgent demand for novel therapeutic approaches. Developing new combination therapies using agents that do not share resistance pathways and have acceptable safety profiles represents a potential strategy to improve outcomes of patients. Plocabulin (PM060184, PharmaMar, Madrid, Spain) is a novel tubulin-binding agent, originally isolated from the marine sponge *Lithoplocamia lithistoides*, and is now obtained by total synthesis [1].

Plocabulin binds tubulin with high affinity, reducing its polymerization and depolymerization to a similar extent and thereby inhibiting microtubule (MT) dynamicity [2–4]. It binds to a site on β-tubulin different from that of vinca alkaloids, eribulin, or taxanes and induces MT depolymerization through a distinct mechanism [2, 4]. It binds to the maytansine site at the longitudinal interface between tubulin dimers and exerts a hinge-like effect that disrupts normal MT assembly [5]. It has also shown antitumor activity in xenograft models expressing the P-glycoprotein (P-gp) multidrug efflux pump, which leads to resistance to vinorelbine and paclitaxel [3]. Plocabulin has strong antiproliferative, and antiangiogenic effect in vitro, and showed cytotoxicity against a broad panel of solid human tumor types [3, 6]. It has potent preclinical anti-cancer activity [7], especially in epithelial ovarian cancer (EOC) [8], gastrointestinal stromal tumor (GIST) [9], colorectal cancer [10], and soft tissue sarcoma [11] models.

Clinical development of plocabulin has included 2 single-agent phase 1 clinical trials. In the first-in-human trial (EudraCT 2010–021855-15), plocabulin was administered intravenously (i.v.) over 10 min at a starting dose of 1.3 mg/m^2^/day on Days 1, 8 and 15 every 4 weeks (q4wk) [12]. The maximum tolerated dose (MTD) was 14.5 mg/m^2^; however, no recommended dose (RD) was confirmed, because frequent dose delays and omissions resulted in low relative dose intensity (66%) at the 12.0 mg/m^2^ expansion cohort. Encouraging antitumor activity- two partial responses (PR) in heavily pretreated non-small cell carcinoma (NSCLC) and cervical carcinoma were observed and the main dose-limiting toxicity (DLT) was grade 3 peripheral sensory neuropathy (PSN). Increasing the duration of infusion from 10 min to 3 h (at 12 mg/m^2^) did not affect compliance or toxicity. Therefore, concerns about its safety remained unresolved at the time. The second phase 1 trial (NCT01299636) compared two different dosing schedules of plocabulin: single dose *vs.* split dosing. In the first schedule, plocabulin was delivered as a 10-min i.v. infusion on Days 1 and 8 every 3 weeks (q3wk) and RD was established at 9.3 mg/m^2^. A similar toxicity profile was observed in this trial, with the most frequent DLT being grade 3 PSN with transient symptoms that involved hands and feet [13]. Common plocabulin-related adverse events (AEs) were mild or moderate and comprised alopecia, fatigue, nausea, vomiting, abdominal pain, and PSN. In the second schedule, based on preclinical data that suggested similar antitumor activity with split dosing over several days compared to a single dose administration, plocabulin was administered on days 1–3 and 15–17 q4wk. Patients were treated at 2 DLs (4 and 5 mg/m^2^); RD was determined to be 4 mg/m^2^. Only 1 patient (n = 14) experienced a DLT of grade 3 bowel obstruction. Common plocabulin-related related laboratory abnormalities showed a similar profile compared to the weekly infusion schedule (Days 1, 8 and 15 q4wk) along with grade 3 vomiting, peripheral motor neuropathy (PMN) and tumor pain. The split dosing schedule overcame the main DLT (PSN) of the weekly schedule, but did not allow for further dose escalation [14]. Based on the above findings, weekly administration of plocabulin over 10 min on days 1 and 8 q3wk was selected for further development.

Gemcitabine is a nucleoside analog with a wide range of activity in various cancers [15]. The standard gemcitabine dose is 800–1000 mg/m^2^ over 30 min weekly [16], and most common toxicity is myelosuppression (mainly low platelet and red blood cell counts, while neutrophils are less affected). Plocabulin has been shown to have synergistic effects with gemcitabine in preclinical studies. In a xenograft pancreatic cancer model of mice, plocabulin alone had a strong anti-tumor effect, while gemcitabine alone had a weaker effect. The analysis by the median-effect principle of the antitumor effect induced by treatments resulted in a combination index (CI) value ≤ 0.1, which suggested a very strong synergistic effect for the combination of both drugs (Supplementary Fig. 1).

Given that the clinical toxicity of each drug is non-overlapping, we launched a combination phase 1 clinical trial of plocabulin and gemcitabine in patients with advanced solid tumors.

## Patients and methods

### Animal studies (plocabulin + gemcitabine combination)

Four-six-week-old athymic *nu/nu* female mice (Harlan Laboratories) were subcutaneously implanted with SW1990 cell suspension (5X10^6^ cells) in Matrigel (1:1). Mice bearing tumors of cancer volume of 150 mm^3^ were then included in the in vivo experiment and allocated to one of 10 groups (n = 6 per group): placebo; plocabulin at 0.75 of its maximum tolerated dose (MTD; 16 mg/kg); plocabulin at 0.5MTD; plocabulin at 0.25MTD; gemcitabine at 0.75MTD (MTD 180.0 mg/kg); gemcitabine at 0.5MTD; gemcitabine at 0.25MTD; plocabulin-gemcitabine at 0.75 + 0.75MTD; plocabulin-gemcitabine at 0.50 + 0.50MTD; and plocabulin-gemcitabine at 0.25 + 0.25MTD. Treatments were given as single intravenous dose.

Tumor volume was recorded two or three times per week starting from the first day of treatment (day 0). Treatment-induced antitumor activity was then determined by percentage (%) of the change in tumor size for treated (T) and placebo (C) groups (ΔT/ΔC). The fraction affected (Fa) by the treatment was calculated as 1-T/C and the combination index (CI) was determined by the CI-isobol method [17] using CompuSyn software v1.0 (ComboSyn Inc.).

### Clinical investigation

This study was conducted at centers in the U.S. and Spain in accordance with the principles of the World Medical Association Declaration of Helsinki, the International Conference of Harmonization, and all applicable local guidelines and regulations on good clinical practice, after receiving required approvals by appropriate research and ethic (scientific and institutional review boards) committees. All the patients signed a written informed consent before any study procedures were conducted.

### Eligibility criteria

All eligible patients were aged ≥ 18 years with histologically/cytologically confirmed diagnosis of selected advanced solid tumors that had progressed on standard therapy or for which standard therapy did not exist, with an Eastern Cooperative Oncology Group (ECOG) performance status (PS) score ≤ 1, recovered from previous toxicities to grade ≤ 1; and with adequate hematological, renal, hepatic and cardiac (ejection fraction) function.

Patients were excluded if they had neuroendocrine, carcinoid, small cell and sarcoma histology subtypes; had received any other antitumor including radiotherapy within 3 weeks or any investigational or biological drugs (excluding monoclonal antibodies) within 4 weeks before the first plocabulin infusion; prior treatment with oxaliplatin, gemcitabine (in metastatic setting) and radiation therapy (RT) > 35% of bone marrow; ≥ grade 2 current or history of peripheral sensory or motor sensitivity; had progressive or symptomatic brain or leptomeningeal metastases; were pregnant or lactating; or had an increased cardiac risk.

### Study design and treatment

This was a dose-escalation phase 1 study to identify DLTs and determine the MTD and the RD for phase 2 trials of plocabulin in combination with gemcitabine both given intravenously (i.v.) on days 1 (D1) and D8 in a 3-week cycle. Plocabulin was provided by PharmaMar as a sterile lyophilized powder concentrate for solution in a strength of 15 mg (active moiety) vials. Before use, the vials were reconstituted with 6 mL of water for injection (2.5 mg/mL). Reconstituted vials were further diluted with 5% dextrose solution for infusion and kept under light protection. Commercially available presentations of vials of i.v. gemcitabine were used. Gemcitabine was infused administered i.v. over 30 min followed by plocabulin over 10 min. Patients received antiemetic prophylaxis with steroids and 5-HT3 antagonists prior to any infusion. Both drugs were administered until disease progression, unacceptable toxicity, patient refusal/non-compliance, treatment delay > 1 week due to treatment-related toxicity, > 2 dose reductions or intercurrent illness precluding safe participation.

### Dose escalation and dose-limiting toxicities

The starting dose of plocabulin was 6.0 mg/m^2^ (i.e., 64% of the RD for this schedule) based on prior Phase 1 single-agent experience [12]. The starting doses for gemcitabine were 800 mg/m^2^, and 1000 mg/m^2^, with provisions for intermediate doses in the event of gemcitabine-related toxicity after agreement between investigator and sponsor. The study followed a classical 3 + 3 design with 3 fully evaluable patients treated at each DL with each patient fully evaluable during a 3-week period prior to further escalation. The MTD was the lowest DL at which more than one third of patients had DLTs whereas the RD was the highest DL at which one third or less of patients had DLTs. Once a dose had been defined as the RD, it was to be confirmed in an expansion cohort of at least 12 evaluable patients in selected tumor types.

DLT were determined in Cycle 1 and were defined as: grade 4 neutropenia lasting > 3 days, febrile neutropenia/neutropenic sepsis, grade 4 thrombocytopenia, grade 3 thrombocytopenia with major bleeding requiring platelet transfusion; grade 3 alanine aminotransferase (ALT) and/or aspartate aminotransferase (AST) elevation > 7 days, or any grade 4 ALT/AST elevation, grade 2 treatment-related ALT/AST elevation with ≥ 2 × upper limit of normal (ULN) total bilirubin with normal alkaline phosphatase (AP); grade ≥ 3 nonhematological toxicity (excluding nausea/vomiting, untreated grade 3 diarrhea lasting < 24 h, grade 3 asthenia lasting < 5 days, hypersensitivity reactions and non-clinically relevant isolated biochemical abnormalities), or treatment delay > 7 days due to toxicity.

### Study assessments

Medical history, physical examination, ECOG PS score, vital signs, laboratory tests evaluating renal, liver and hematological function, appropriate tumor marker, electrocardiogram (ECG) and left ventricular ejection fraction (LVEF) were assessed at baseline. Patients were evaluated weekly while on treatment. LVEF was assessed every 2 cycles. On treatment ECGs were performed if clinically indicated. Adverse events (AEs) were assessed throughout treatment, and laboratory values that reached grade ≥ 3 were re-assessed at least every 2–3 days or daily for selected toxicities until recovery. AEs and laboratory abnormalities were graded (G) according to the National Cancer Institute Common Terminology Criteria for Adverse Events (NCI-CTCAE) v.4. Tumor assessments were evaluated radiographically using RECIST v.1.1 [18] every 6 weeks by computed tomography (CT) scans for the first 4 cycles, and every 9 weeks thereafter. Patients with GIST were assessed through positron emission tomography (PET)-CTs using CHOI [19] and/or European Organization for Research and Treatment of Cancer (EORTC) metabolic response criteria, or by evaluation of serum tumor markers if applicable. Assessment of serum tumor markers was repeated every 3 weeks, if elevated at baseline.

### Pharmacokinetic and pharmacogenetic analyses

Blood samples for PK assays were collected on D1 and D2 of Cycle 1 at all DLs for plasma concentrations of plocabulin, gemcitabine and its metabolite 2’,2’-difluorodeoxyuridine (dFdU). PK was evaluated by standard non-compartmental analysis (NCA) as part of secondary endpoints. Further, to explore factors that may help explain individual variability in main PK parameters, the presence or absence of germline mutations or polymorphisms was analyzed in leukocyte DNA extracted from a pharmacogenetic blood sample (PG), collected once during the study.

## Results

### Human-derived cell line xenograft study

Six mice were treated in each of the ten conditions. Rapid tumor growth was observed in the control mice, and all control mice were sacrificed on Day 19 from start of drug intervention. As single agents, plocabulin and gemcitabine exhibited a similar degree of tumor growth control. The mice that received both drugs clearly had the best outcome, with some mice showing complete tumor regressions (CI < 0.1). All mice were sacrificed and euthanized when the tumor volume reached 2,000 mm^3^ or at the end of the experiment at 4 weeks (Supplementary Fig. 1, data shown for 4 conditions).

### Patient characteristics

The study enrolled 57 patients (Table 1); 42 (73.7%) were females; 26 (45.6%) were white and median age was 62 years (range, 25–80 years). Most patients had ECOG PS score of 1 (n = 42, 73.7%). The most common primary tumor type was non-small cell lung cancer (NSCLC, n = 18, 31.6%), and EOC (n = 13, 22.8%). All patients had received prior systemic anticancer therapy, with a median of 3 prior lines (range, 1–7 lines) and 4 agents (range, 2–9 agents). Seventeen patients (29.8%) received ≥ 4 lines of prior anticancer therapies each. Non-medical therapy included surgery (n = 38, 66.7%) and radiotherapy (n = 36, 63.2%).

**Table 1:** Baseline characteristics of patients (n = 57)

			**All patients** (n = 57)
Gender	Male		15 (26.3)
	Female		42 (73.7%)
Age (median)			62 (25–80)
Race	White		26 (45.6%)
	Black or African-American		12 (21.1%)
	Asian		1 (1.8%)
	Other/Unknown		18 (31.6%)
Primary tumor type	NSCLC		18 (31.6%)
	Epithelial ovarian cancer		13 (22.8%)
	Cervical		8 (14%)
	Endometrial		5 (8.8%)
	MBC		4 (7.0%)
	GIST		3 (5.3%)
	HNSCC		3 (5.3%)
	GCT		2 (3.5%)
	CUP		1 (1.8%)
ECOG PS score:	0		15 (26.3%)
	1		42 (73.7%)
Metastatic sites	Lymph nodes		40 (70.2%)
	Lung		38 (66.7%)
	Liver		26 (45.6%)
	Peritoneum		19 (33.3%)
Prior systemic anticancer therapy	Median no. of prior lines (range)		3 (1–7)
	Patients with ≥ 4 prior lines (%)		29.8%
Most common anticancer agents	Taxanes	Paclitaxel Docetaxel	42 (73.7%) 12 (21.1%)
	Platinum compounds	Carboplatin Cisplatin	37 (64.9%) 26 (45.6%)
	Monoclonal antibodies	Bevacizumab	18 (31.6%)
	Folic acid analogues	Pemetrexed disodium	14 (24.6%)
	Other therapies	Surgery Radiotherapy	38 (66.7%) 36 (63.2%)

*CUP* cancer of unknown primary site, *ECOG* Eastern Cooperative Oncology Group, *GCT* germ cell tumor, *GIST* gastrointestinal stromal tumor, *HNSCC* head and neck squamous cell carcinoma, *MBC* metastatic breast cancer, *NSCLC* non-small cell lung cancer, *PS* performance status

### Safety, DLTs, MTD, and RD

Of the 57 enrolled patients, 55 patients received at least one dose of study drug. Patients received a median of 3 cycles (range, 1–16 cycles) of therapy. Most patients (n = 32, 58.2%) at all DLs discontinued treatment due to disease progression. Ten patients (18.2%) refused further treatment with the combination. Five patients (9.1%) discontinued due to treatment-related AEs [PSN (G1-1; G2-2; G3-1)]; and grade 3 pneumonitis (n = 1)]. Four patients (7.3%) discontinued following a decision by the investigator in the absence of radiologically documented disease progression. Three patients (5.5%) died while on treatment due to disease-related AEs. Finally, one patient (1.8%) discontinued due to a non-treatment-related AE.

All 55 treated patients were evaluated for safety. Most treatment emergent AEs (or with unknown relationship) at all DLs were grade 1 or 2 (Table 2). The most common were fatigue (56.4% of patients), nausea (54.5%), PSN (36.4%), musculoskeletal (32.7%), diarrhea (30.9%), decreased appetite (27.3%), vomiting (27.3%), abdominal pain (16.4%), and constipation (14.5%). Treatment-related grade ≥ 3 AEs comprised fatigue (3 patients), abdominal pain, nausea, vomiting (2 patients each), arthralgia, diarrhea, dyspnea, intestinal obstruction, muscular weakness, neurotoxicity, and pneumonitis (1 patient each). None of these AEs reached grade 4.

**Table 2:** Treatment emergent adverse events and laboratory abnormalities

Dose level	1		2			3		4		5			6			7			8					
Plocabulin mg/m^2^	6		7			7		8		9			9.3			10			10.5					
Gemcitabine mg/m^2^	800		800			1000		1000		1000			1000			1000			1000				Total	
# patients	4		7			4		5		5			9			16			5				55	(% patients)
Grade	< 2	3	< 2	3	4	< 2	3	< 2	3	< 2	3	4	< 2	3	4	< 2	3	4	< 2	3	4		50	90.9
Gastrointestinal																								
Diarrhea	1		2			1				1			2			7	1		2				17	30.9
Dyspepsia	1															1							2	3.6
Nausea	2		4					2		4			4			9	2		3				30	54.5
Vomiting			2				1	1		1	1		2			5			2				15	27.3
Abdominal pain							1						2			4	1		1				9	16.4
Constipation													2			4			2				8	14.5
Anorexia	1					1		2		2			1			7			1				15	27.3
Hematochezia													1										1	1.8
Intestinal obstruction	1		1			1		1		2			4			6			3				19	34.5
Dry mouth																2							2	3.6
Neurotoxicity																								
PSN	1		1	1		1		1		2			4			6		3					20	36.4
ANS-mediated bowel obstruction				1																			1	1.8
Constitutional																								
Fatigue	2		3			4		2		3	1		5			7	1		2	1			31	56.4
Pyrexia	1					1		2								1							5	9.1
Weight decreased																2							2	3.6
Headache																1			1				2	3.6
Alopecia			1					1											1				3	5.5
Others																								
Urinary tract infection	1																						1	1.8
Musculoskeletal	3					2					1					7			4	1			18	32.7
Rash maculo- papular								1															1	1.8
Skin disorder						1										4							5	9.1
Dyspnea									1				1			1							3	5.5
Pneumonitis																				1			1	1.8
Palpitation								1															1	1.8
Hematologic																								
Anemia	2		3	3		3		5		3	1		5	4		7	8		3	2			49	89.1
Neutropenia	1	2	1	1		3		3	2	3		1	3	1		3	4	1	1	1	1		32	58.2
Leukopenia	2	1	3		1	3		5		3	1		8	1		11	2		2	2			45	81.8
Lymphopenia	1	1	2	2		1	1	3	1	3			7	1		8	2	1	3	1	1		39	70.9
Thrombocytopenia	3		4	1		2	1	4		5			6		1	10	5		3	1			46	83.6
Biochemical																								
ALT increased	1		5			3		3		3			4	1		6	1		5				32	58.2
AST increased			5			3		1	1	2			5			6	1		3				27	49.1
AP increased	1		4			1		1		3			4	1		6	1		1	1			24	43.6
Bilirubin increased															1	1			1				3	5.5
Creatinine increased	1		3			1		3		3			4			6			1				22	40.0

*ALT* alanine aminotransferase, *ANS* autonomic nervous system, *AP* alkaline phosphatase, *AST* aspartate aminotransferase, *PSN* peripheral sensory neuropathy

Hematological abnormalities regardless of relationship were present at all DLs. Severe myelosuppression comprised grade 3 anemia (34.6%), grade 3/4 neutropenia (26.9%), and grade 3/4 thrombocytopenia (17.3% of patients). Hematological toxicity was the cause of most cycle delays and dose omission at all DLs, but did not result in treatment discontinuations.

Most biochemical abnormalities at all DLs were grade 1 or 2, regardless of relationship. Severe biochemical abnormalities were found in only one (1.9%) to three (5.8%) patients each and were mostly grade 3; the only one to reach grade 4 was bilirubin increase in one patient who had concomitant disease-related grade 3 ALT increase and disease progression in the liver. No biochemical abnormalities resulted in cycle delays, dose omissions or dose reductions.

At all DLs, dose delays were observed in 23 patients (54.8% of 42 patients who received at least 2 cycles each) in 54 cycles (30.3% of 178 cycles susceptible of delay). Dose omissions of either gemcitabine or plocabulin were carried out in 24 patients (47.1% of 51 patients susceptible of dose omission) and 53 cycles (23.2% of 228 cycles susceptible of dose omission). Gemcitabine dose reductions occurred in 8 patients (19.0% of 42 patients) and plocabulin dose reductions also in 8 patients (19.0% of 42 patients).

DLTs were observed at 4 DLs (Table 3): two grade 3 neurotoxicity’s categorized as grade 3 PSN in one patient at DL2 (n = 6, 16.7%) and grade 3 bowel obstruction [autonomic nervous system (ANS) dysregulation] in one patient at DL8 (n = 5, 20%), grade 4 thrombocytopenia in one patient at DL6 (n = 7, 14%) and grade 3 abdominal pain in one patient at DL7 (n = 13, 7.7%). Further dose escalation was stopped following the finding at DL8 of treatment-related Cycle 1 Day 8 dose omissions and reductions from Cycle 2 onwards in 3 and 2 patients, respectively.

**Table 3:** DLTs and dose escalation

Dose level	Dose		No. of patients with DLTs / no. of evaluable patients		DLT
	Plocabulin (mg/m^2^)	Gemcitabine (mg/m^2^)			
1	6.0	800	0/3		
2	7.0	800	1/6		Grade 3 PSN^a^
3	7.0	1000	0/3		
4	8.0	1000	0/3		
5	9.0	1000	0/4		
6	9.3	1000	1/7		Grade 4 thrombocytopenia^b^
7	10.0	1000	Overall: 1/13	0/7	
				Cohort expansion: 1/6	Grade 3 abdominal pain^a^
8 (MTD)	10.5	1000	1/5		Grade 3 neurotoxicity (ANS-mediated bowel obstruction)^a^

*PSN* peripheral sensory neuropathy, *ANS* autonomic nervous system, *DLT* dose-limiting toxicity, *MTD* maximum tolerated dose

^a^ Related to plocabulin

^b^ Related to both plocabulin and gemcitabine

The MTD for a combination of gemcitabine given i.v. over 30 min followed by plocabulin given i.v. over 10 min, both on Day 1 and Day 8 q3wk, in patients with advanced solid tumors was defined at DL8 (plocabulin 10.5 mg/m^2^ plus gemcitabine 1000 mg/m^2^). Patient accrual was discontinued before an RD had been formally determined because of the narrow therapeutic index, toxicity profile, and limited antitumor activity of the combination.

Eight patients (14.5%) died during this study. Five deaths (5/55; 9.1%) were due to disease progression, and 3 (3/55; 5.4%) deaths were due to disease-related AEs (grade 4 sepsis secondary to aspiration pneumonia, grade 5 respiratory arrest and grade 5 dyspnea). All 3 patients who died due to disease-related AEs had tumor lesions in the lungs at baseline.

### Pharmacokinetics

Mean maximum plasma concentration (C_max_), area under the concentration–time curve, and half-life for plocabulin at the DL immediately below the MTD (DL7: plocabulin 10.0 mg/m^2^ plus gemcitabine 1000 mg/m^2^) in Cycle 1 were 665 μg/L, 430 h*mg/L and 4 h, respectively (Supplementary Table 1a). PK parameters for gemcitabine or dFdU at all DLs were in line with reference values from the literature (Supplementary Table 1b) [20–22].

### Efficacy outcomes

**Fig. 1:**
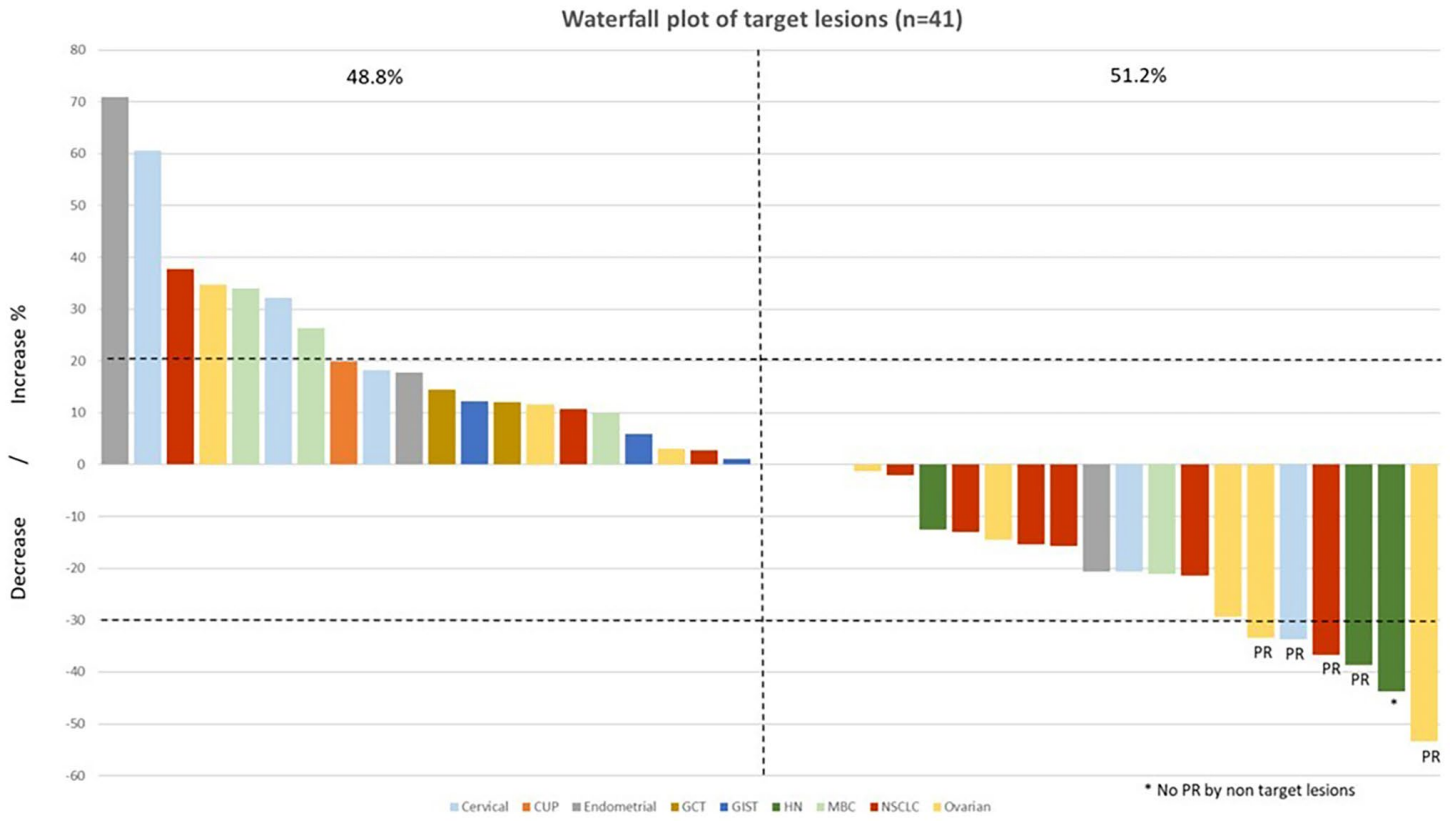
Waterfall plot of best response among the 41 patients who had measurable lesions at baseline. Each tumor type is color-coded. An upward pointing bar that exceeds 20% represents progressive cancer, a downward bar exceeding 30% indicates a partial response, and the rest of bars are stable diseases. One patient had a partial response of target lesions, but not of non-target lesions. One patient experienced a complete response (not shown). CUP, cancer of unknown primary site; GCT, germ cell tumor; GIST, gastrointestinal stromal tumor; HN, head and neck squamous cell carcinoma; MBC, metastatic breast cancer; NSCLC, non-small cell lung cancer; PR, partial response

**Fig. 2:**
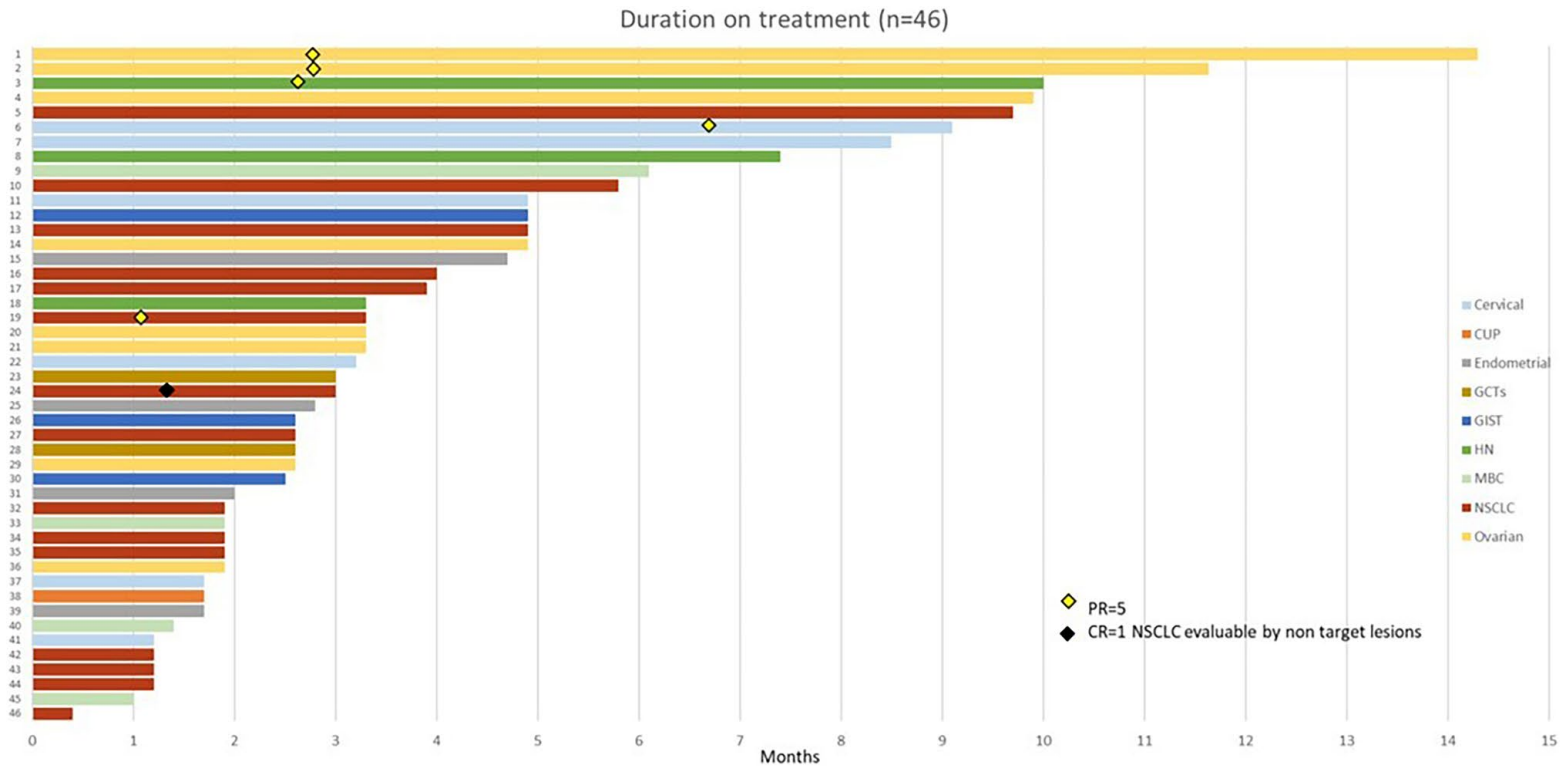
Swimmer’s plot of 46 patients with color codes for each tumor type. The time of first documented response is indicated by a diamond plot. CR, complete response; CUP, cancer of unknown primary site; GCT, germ cell tumor; GIST, gastrointestinal stromal tumor; HN, head and neck squamous cell carcinoma; MBC, metastatic breast cancer; NSCLC, non-small cell lung cancer; PR, partial response

Forty-six patients treated at all DLs were evaluable for efficacy. Overall, 6 patients had a response- 1 Complete response (CR) and 5 partial responses (PR) (overall response rate [ORR] = 13%) and 12 patients had stable disease (SD) ≥ 4 months. Clinical benefit (ORR plus SD ≥ 4 months) was observed in 18 of 46 (39.1%) evaluable patients (Supplementary Table 2, Figs. 1–2). Clinical benefit was observed in NSCLC (n = 6; one CR, one PR, and 4 SD ≥ 4 months), gynecological tumors (n = 5; one PR and 4 SD ≥ 4 months), EOC (n = 4; 2 PRs and 2 SD ≥ 4 months), head and neck cancer (n = 2; one PR and one SD ≥ 4 months), and breast cancer (n = 1; one SD ≥ 4 months). In 13 patients with clinical benefit, the progression free survival (PFS) values achieved with the combination were longer than the time to progression (TTP) values achieved with the last prior therapy, including patients with gynecological tumors (n = 4), EOC (n = 4), NSCLC (n = 2), head and neck cancer (n = 2), and breast cancer (n = 1).

## Discussion

Patients with advanced treatment refractory solid tumors lack sufficient therapies that hold the potential to improve outcomes. To this end, marine derived chemicals have been discovered to be a great resource. Plocabulin is a novel marine derived MT destabilizing agent with potent antineoplastic activity, bearing unique structural and mechanistic features. Given that P-gp is responsible for paclitaxel resistance, and that plocabulin is active in P-gp-expressing tumors[3] this may suggest a mechanistic basis for activity in paclitaxel-resistant tumors that was clearly observed in this trial.

A prior single-agent phase 1 study with plocabulin showed that the drug was both safe and effective in patients with advanced solid tumors, and identified PSN as the main DLT[12]. Interestingly, a RD for single-agent plocabulin could not be defined in this study because patients did not tolerate well the administration of several cycles of the MTD (14.5 mg/m^2^ as a 10-min i.v. infusion on Days 1, 8 and 15 every 4 weeks). In the current combination study of plocabulin with gemcitabine, neurotoxicity (PSN) was once more the first DLT observed. Further, the bowel obstruction at the MTD in the present study suggested an ANS-mediated neurotoxicity as well. Of note, the overall incidence of neurotoxicity (PSN and bowel obstruction) in the combination study was lower than in the single-agent study, likely attributable to the overall lower doses of plocabulin administered. For example, in the single-agent plocabulin study, 3 of 44 (6.8%) treated patients (2 at the MTD of 14.5 mg/m^2^ and one at 12.0 mg/m^2^) experienced grade 3 neurotoxicity, while in the combination study, only one of 55 (1.8%) treated patients (at plocabulin 7.0 mg/m^2^ plus gemcitabine 800 mg/m^2^) did so. Lessons learnt from one trial when applied in real time to subsequent trials is the ideal sequence of drug development. This is especially critical when dealing with tubulin interacting drugs, with almost universal neurotoxicity, and with an almost assured prior exposure to one of such agents. Strategies for mitigating and managing neurotoxicity will therefore be crucial for further development of plocabulin. Similarly, at all DLs several treatment-related AEs commonly found with single-agent plocabulin were less frequent with the combination.

By far, hematological toxicities were the most frequently observed in this combination study. This was expected, given that both plocabulin and gemcitabine are myelosuppressive. Anemia (grade 3 only) was the most common severe hematological toxicity, followed by grade ¾ neutropenia and grade ¾ thrombocytopenia. There was an increase in the incidence of thrombocytopenia at all DLs with the combination as compared to single-agent plocabulin (88.5% *vs.* 41%, respectively), a clear effect of gemcitabine. Encouragingly, although hematological toxicities were responsible for most dose delays and omissions, they did not result in any treatment discontinuations. Similar to the single-agent trial [12], fatigue was the most common non-hematological toxicity at all DLs with the combination (any grade, 56.4% vs. 77% with single-agent plocabulin). Other common toxicities observed with the combination (diarrhea, nausea, anorexia, and abdominal pain) were easily manageable.

Despite no formal RD determined for the combination, the finding of DLTs in one of 13 patients treated at the second-highest DL evaluated (plocabulin 10.0 mg/m^2^ plus gemcitabine 1000 mg/m^2^) suggests that this is perhaps the appropriate dose for this combination.

The mean clearance and C_max_ observed for plocabulin at all DLs were similar to the mean values found for these parameters in previous phase 1 and 2 studies(1–3), thereby suggesting that gemcitabine has no major effects on the PK profile of plocabulin. The PK parameters of gemcitabine and dFdU were in line with values reported in the literature. Thus, no evidence of major drug-drug interaction between plocabulin and gemcitabine was observed.

When evaluating combination therapies, it is critical to carefully consider whether the combination is simply additive or truly synergistic. Preclinical studies clearly demonstrated the synergistic effect of the combination of plocabulin and gemcitabine, therefore providing a strong rationale for the current trial. The data also suggest that the combination is superior to single agent therapy, as is evidenced by a higher response rate (RR = 13.3%) and a much-improved disease control rate (40%).

Clinical benefit was observed in several patients, and among them, objective responses were experienced by 6 patients. Interestingly, these comprised patients with tumor types that are known to be responsive to gemcitabine, such as NSCLC and EOC. Among patients with NSCLC, 2 of 6 enjoyed a response, and 2 of 4 patients with EOC did so. All the responding patients had prior therapy with platinum compounds or taxanes, revealing a role for plocabulin in chemotherapy refractory patients. The finding of 2 PRs among 6 evaluable patients with EOC is striking. Single-agent gemcitabine has historically yielded a RR ranging from 11 to 22%, at best [23, 24]. While novel agents have received approval for treatment of EOC, there is still a clear need for safer and more effective therapeutic options. In this regard, the combination of plocabulin and gemcitabine may offer such a novel and safe option for these patients. Participation of patients with EOC in therapeutic trails with novel agents is thus encouraged, as it clearly results in clinical benefit [25].

## Supplementary Information

Below is the link to the electronic supplementary material.

Supplementary Table 1: Details of pharmacokinetic parameters at each dose level of plocabulin (Table 1a), gemcitabine (Table 1b) and 2,2-difluorodeoxyuridine (dFdU) (Table 1c). (DOCX 75.6 KB)

Supplementary Table 2: Characteristics of patients with complete or partial response to plocabulin plus gemcitabine. Of the 45 evaluable patients, 6 experienced a partial (5) or complete (1) response. (DOCX 34.5 KB)

Supplementary Figure 1: Tumor volumes of xenograft model of pancreas cancer cell line SW-1190 in nude mice. The combination index of 0.06 indicates synergy between plocabulin and gemcitabine. CI, combination index; MTD, maximum tolerated dose; q7d, every seven days. (JPG 136 KB)

## Data Availability

Who can access the data: Anyone requesting data. Types of analyses: Any other analysis. Mechanisms of data availability: Signed DUA. Any additional restrictions: We are unable to share the data via a publicly available system due to privacy concerns. If an individual reader wishes to obtain information on a specific aspect of the data, we can reach out to our institution policy and attempt to obtain consent to share patient level data. We can share the statistical analysis information.

## Declarations

### Presentations

Presented in part at the Annual Meetings of the European Society of Medical Oncology (ESMO) in 2023, Madrid, Spain.
