# Supplementary material for: A phase I safety and efficacy clinical trial of plocabulin and gemcitabine in patients with advanced solid tumors
Source: Invest New Drugs. 2024 Aug 3;42(5):481–91. doi: 10.1007/s10637-024-01458-8 (PMC11625058; doi:10.1007/s10637-024-01458-8)
Supplement: Supplementary file 1 — Details of pharmacokinetic parameters at each dose level of plocabulin (Table 1a), gemcitabine (Table 1b) and 2,2-difluorodeoxyuridine (dFdU) (Table 1c). (DOCX 75.6 KB) [file 10637_2024_1458_MOESM1_ESM.docx]

Supplementary Table 1a: Plasma pharmacokinetic profile for plocabulin by cycle.

| **Cycle** | **Plocabulin**  **dose level (mg/m²)** |  | **Cmax (µg/L)** | **AUC**  **(h*µg/L)** | **CL**  **(L/h)** | **HL**  **(h)** | **Vss (L)** |
| --- | --- | --- | --- | --- | --- | --- | --- |
| **1** | **6.0** | **n** | 4 | 4 | 4 | 4 | 4 |
|  |  | **Mean** | 134.48 | 118.49 | 95.76 | 4.08 | 378.70 |
|  |  | **SDev** | 41.78 | 67.82 | 35.28 | 1.86 | 258.61 |
|  | **7.0** | **n** | 11 | 11 | 11 | 11 | 11 |
|  |  | **Mean** | 302.87 | 189.41 | 74.98 | 3.43 | 202.23 |
|  |  | **SDev** | 198.67 | 70.27 | 32.86 | 1.21 | 147.45 |
|  | **8.0** | **n** | 5 | 3 | 3 | 3 | 3 |
|  |  | **Mean** | 263.50 | 665.01 | 42.02 | 3.21 | 115.64 |
|  |  | **SDev** | 174.52 | 713.07 | 28.97 | 0.91 | 81.13 |
|  | **9.0** | **n** | 5 | 5 | 5 | 5 | 5 |
|  |  | **Mean** | 345.14 | 408.58 | 58.78 | 4.96 | 200.62 |
|  |  | **SDev** | 237.14 | 313.77 | 28.98 | 1.35 | 116.46 |
|  | **9.3** | **n** | 9 | 8 | 8 | 8 | 8 |
|  |  | **Mean** | 273.39 | 424.41 | 46.98 | 5.05 | 199.17 |
|  |  | **SDev** | 181.84 | 227.00 | 22.30 | 1.14 | 103.74 |
|  | **10.0** | **n** | 16 | 16 | 16 | 16 | 16 |
|  |  | **Mean** | 664.81 | 439.84 | 48.93 | 4.13 | 130.67 |
|  |  | **SDev** | 524.18 | 253.41 | 20.22 | 1.31 | 57.33 |
|  | **10.5** | **n** | 5 | 5 | 5 | 5 | 5 |
|  |  | **Mean** | 499.80 | 465.23 | 42.60 | 4.85 | 151.69 |
|  |  | **SDev** | 119.26 | 143.19 | 12.45 | 0.52 | 79.50 |
| **2** | **10.0** | **n** | 4 |  | 4 | 4 |  |
|  |  | **Mean** | 1071.93 | 556.68 | 41.500 | 3.98 | 93.77 |
|  |  | **SDev** | 911.68 | 266.05 | 35.28 | 4.59 | 71.92 |

AUC, area under the concentration-time curve from time zero to infinity; CL, clearance; C_max_, maximum plasma concentration; HL, terminal half-life; n, number of patients; SDev, standard deviation; V_ss_, volume of distribution at steady-state.

Supplementary Table 1b: Plasma PK parameters for gemcitabine by dose.

| **GEM**  **dose level**  **(mg/m²)** |  | **Cmax (mg/L)** | **AUC**  **(h*mg/L)** | **CL**  **(L/h)** | **HL**  **(h)** | **Vss**  **(L)** |
| --- | --- | --- | --- | --- | --- | --- |
| **800** | **n** | 11 | 7 | 7 | 7 | 7 |
|  | **Mean** | 8.97 | 5.36 | 251.04 | 0.28 | 95.89 |
|  | **SDev** | 5.69 | 1.13 | 52.96 | 0.05 | 21.57 |
| **1000** | **n** | 44 | 43 | 43 | 43 | 43 |
|  | **Mean** | 15.87 | 7.8 | 296.65 | 0.32 | 128.15 |
|  | **SDev** | 10.84 | 4.24 | 150.91 | 0.16 | 93.09 |

AUC, area under the concentration-time curve from time zero to infinity; CL, clearance; C_max_, maximum plasma concentration; GEM, gemcitabine; HL, terminal half-life; n, number of patients; SDev, standard deviation; V_ss_, volume of distribution at steady-state.

Supplementary Table 1c: Plasma PK parameters for dFdU by dose.

| **GEM**  **dose level**  **(mg/m²)** |  | **Cmax (mg/L)** | **AUC**  **(h*mg/L)** | **CL**  **(L/h)** | **HL**  **(h)** | **Vss**  **(L)** |
| --- | --- | --- | --- | --- | --- | --- |
| **800** | **n** | 11 | 7 | 7 | 7 | 7 |
|  | **Mean** | 27.01 | 289.73 | 4.68 | 12.56 | 74.61 |
|  | **SDev** | 7.48 | 61.31 | 0.76 | 4.83 | 26.92 |
| **1000** | **n** | 44 | 43 | 43 | 43 | 43 |
|  | **Mean** | 39.46 | 336.93 | 5.72 | 10.18 | 66.58 |
|  | **SDev** | 5.65 | 94.48 | 1.90 | 2.95 | 22.51 |

AUC, area under the concentration-time curve from time zero to infinity; CL, clearance; C_max_, maximum plasma concentration; CV, coefficient of variation (%); dFdU, 2,2-difluorodeoxyuridine; GEM, gemcitabine; HL, terminal half-life; Max, maximum value; Min, minimum value; n, number of patients; SDev, standard deviation; V_ss_, volume of distribution at steady-state.
