# Supplementary material for: A phase I safety and efficacy clinical trial of plocabulin and gemcitabine in patients with advanced solid tumors
Source: Invest New Drugs. 2024 Aug 3;42(5):481–91. doi: 10.1007/s10637-024-01458-8 (PMC11625058; doi:10.1007/s10637-024-01458-8)
Supplement: Supplementary file 2 — Characteristics of patients with complete or partial response to plocabulin plus gemcitabine. Of the 45 evaluable patients, 6 experienced a partial (5) or complete (1) response. (DOCX 34.5 KB) [file 10637_2024_1458_MOESM2_ESM.docx]

**Supplementary Table 2: Characteristics of 6 responding patients (of 45 evaluable patients)**

| # | Age  (years) | Gender | Tumor type | Histology/sub type | Best response | Time on study (days) | No. of prior regimens | Prior regimens | | | |
| --- | --- | --- | --- | --- | --- | --- | --- | --- | --- | --- | --- |
|  |  |  |  |  |  |  |  | 1 | 2 | 3 | 4 |
|  |  |  |  |  |  |  |  |  |  |  |  |
| 1 | 65 | M | Larynx | Squamous cell | PR | 282 | 2 | Car/Cet/5-FU | Pac | . | . |
| 2 | 74 | M | Lung | NSCLC | PR | 106 | 2 | Car/Pac | Nivo | . | . |
| 3 | 59 | F | Ovary | Epithelial | PR | 466 | 4 | Car/Pac | Car/Doxo | Pac/Bev | Doc/Bev |
| 4 | 44 | F | Ovary | Epithelial | PR | 363 | 4 | Car/Pac | Doxo | Car | Cyclo/Bev |
| 5 | 69 | M | Lung | NSCLC | CR | 110 | 1 | Cis/Pem | . | . | . |
| 6 | 55 | F | Cervical | Adenocarcinoma | PR | 269 | 3 | Cis/Pac | Vino | Vino | . |
| 5-FU, 5-fluorouracil; Bev, bevacizumab; Car, carboplatin; Cet, cetuximab; Cis, cisplatin; CR, complete response; Cyclo, cyclophosphamide; Doc, docetaxel; Doxo, doxorubicin; F, female; M, male; Nivo, nivolumab; NSCLC, non-small cell lung cancer; Pac, paclitaxel; Pem, pemetrexed; PR, partial response; Vino, vinorelbine. | | | | | | | | | | | |
