# Supplementary figures and images for: A phase I safety and efficacy clinical trial of plocabulin and gemcitabine in patients with advanced solid tumors
Source: Invest New Drugs. 2024 Aug 3;42(5):481–91. doi: 10.1007/s10637-024-01458-8 (PMC11625058; doi:10.1007/s10637-024-01458-8)

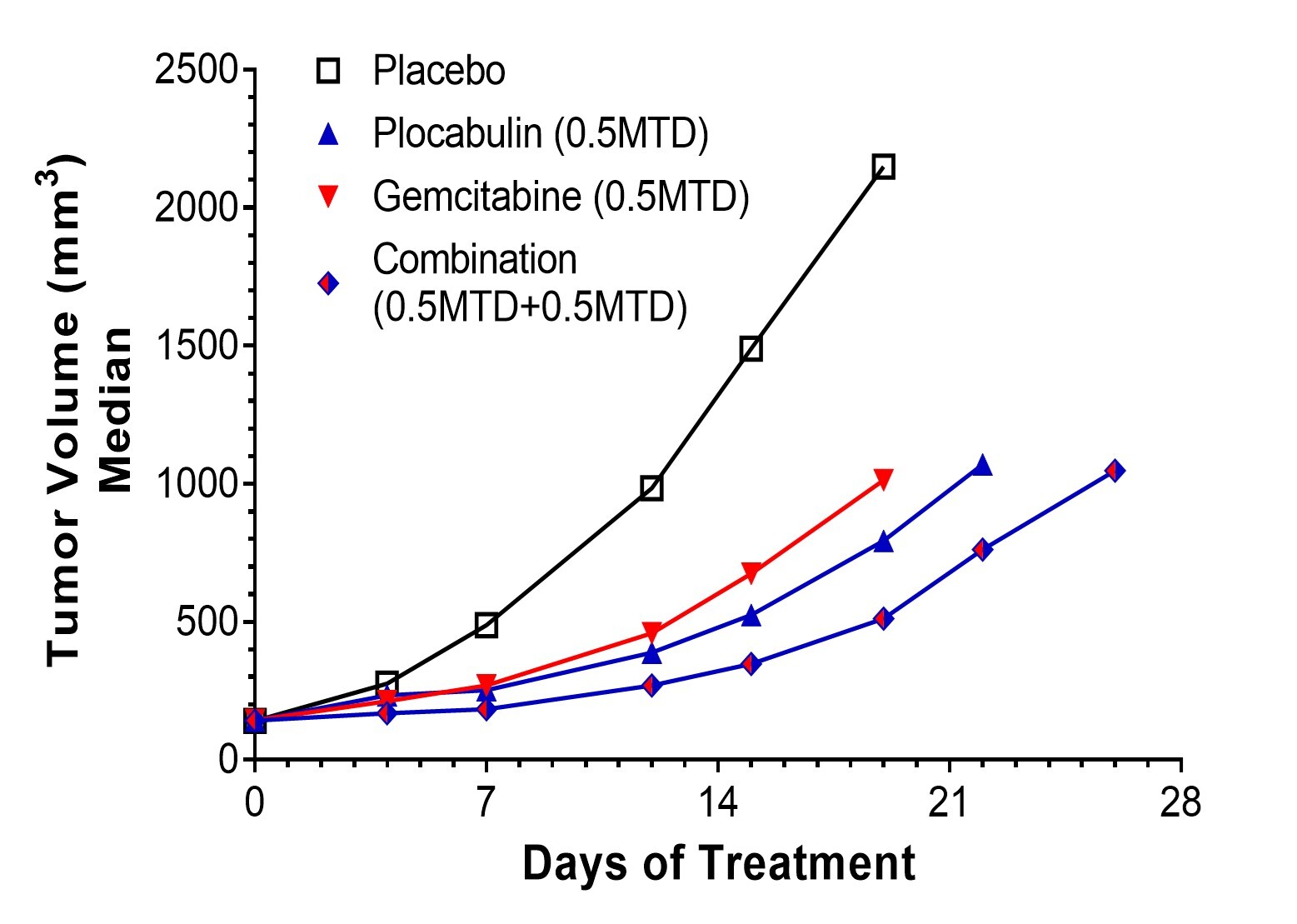

Supplement: Supplementary file 3 — Tumor volumes of xenograft model of pancreas cancer cell line SW-1190 in nude mice. The combination index of 0.06 indicates synergy between plocabulin and gemcitabine. CI, combination index; MTD, maximum tolerated dose; q7d, every seven days. (JPG 136 KB) [file 10637_2024_1458_MOESM3_ESM.jpg]
